# Efficacy and safety of local treatments combined with targeted therapy and immunotherapy versus targeted therapy plus immunotherapy alone in hepatocellular carcinoma: A meta-analysis

**DOI:** 10.1097/MD.0000000000049221

**Published:** 2026-06-12

**Authors:** Shaohua Zhang, Ying Tang, Xinbo Mao, Tao Sheng

**Affiliations:** a Department of Hepatobiliary Surgery, Affiliated Hospital of North Sichuan Medical College, Nanchong, Sichuan, China; b Department of Ultrasound, Affiliated Hospital of North Sichuan Medical College, Nanchong, Sichuan, China.

**Keywords:** hepatic arterial infusion chemotherapy, hepatocellular carcinoma, immune checkpoint inhibitors, meta-analysis, transarterial chemotherapy, tyrosine kinase inhibitors

## Abstract

**Background:**

Beyond surgical intervention, hepatocellular carcinoma is mainly treated with 3 main approaches: transarterial chemoembolization (TACE) or hepatic arterial infusion chemotherapy (HAIC) as local treatment, immune checkpoint inhibitors (ICIs), and tyrosine kinase inhibitors (TKIs). Up to now, clinical experts have not reached a consistent consensus on how to reasonably select combination therapy. Therefore, we performed this research to contrast the clinical outcomes of local treatment plus ICIs and TKIs (Local-ICIs-TKIs) with ICIs and TKIs, aiming to offer a practical reference basis for clinical practice.

**Methods:**

We searched the PubMed, Web of Science, Embase, Cochrane Library, China National Knowledge Infrastructure, and Wanfang databases on the computer, covering literature from their establishment to August 1st, 2025, for (Local-ICIs-TKIs) compared to ICIs and TKIs Clinical Investigation. The primary outcome indicators were treatment-related adverse events, median overall survival, median progression-free survival, objective response rate, and disease control rate. All statistical analyses were conducted using Stata 17 statistical software in this study.

**Results:**

We screened 15 relevant pieces of literature, including 3208 patients. Compared with the ICIs-TKIs group, the Local-ICIs-TKIs treatment enhanced the pooled disease control rate (relative risk = 1.27; 95% confidence interval [CI]: 1.21–1.33) and objective response rate (relative risk = 1.87; 95% CI: 1.71–2.05). The combination therapy individually prolonged the median progression-free survival (hazard ratio = 0.59; 95% CI: 0.47–0.71) and median overall survival (hazard ratio = 0.48; 95% CI: 0.40–0.56). TACE and HAIC significantly elevated the risks of abdominal pain, anorexia, nausea, appetite loss, leukopenia, and liver injury. HAIC exhibited a higher incidence of abdominal pain, whereas TACE was more likely to lead to severe liver injury. The majority of adverse events were mild to moderate, and both interventions demonstrated favorable safety.

**Conclusion:**

In comparison to the foundation of ICIs-TKIs systemic therapy, the addition of TACE or HAIC treatment exhibits good tolerability and can further yield superior clinical responses and prolong survival time for hepatocellular carcinoma patients.

## 1. Introduction

Hepatocellular carcinoma (HCC), as the most prevalent and aggressive form of liver cancer globally, has an annual number of newly diagnosed liver cancer cases in the world that reached 906,000, ranking sixth among malignant tumors.^[1]^ It is projected that the worldwide incidence of HCC will witness a remarkable increase. According to reliable and comprehensive data sourced from the global cancer database for the year 2022,^[2]^ the annual new cases and deaths of HCC in China account for nearly half of the world. The treatment of advanced HCC is still facing a very great challenge. Early HCC development is insidious and progresses rapidly. When diagnosed, a large number of patients are already in an advanced clinical stage and have no chance of surgery.^[3]^ Transarterial chemoembolization (TACE) and hepatic arterial infusion chemotherapy (HAIC) are the common therapy methods for advanced HCC. This local treatment blocks the blood supply to the tumor, playing an important role. However, it induces hypoxia and increases the level of vascular endothelial growth factor in HCC. On the other hand, chemotherapy drugs can damage the patient’s immune system, which is one of the important factors driving tumor growth, progression, recurrence, and metastasis.^[4,5]^ Without chemotherapy, the tyrosine kinase inhibitors (TKIs) are targeted therapy medications that inhibit tumor angiogenesis, primarily used for treating malignant tumors such as primary liver cancers.^[6]^ However, using this kind of treatment alone cannot obviously prolong survival time. immune checkpoint inhibitors (ICIs) are new methods for the treatment of HCC. Programmed cell death protein-1 (PD-1) inhibitors have yielded promising clinical efficacy and safety in patients with advanced HCC^.[7–9]^ Systemic therapy for advanced or unresectable HCC, especially anti-angiogenic drugs combined with immunotherapy, can achieve an objective response rate (ORR) of about 30%, and the median survival time for patients receiving this type of therapy can be as long as 20 months.^[10,11]^ Other research indicated that Camrelizumab combined with apatinib showed promising efficacy and manageable safety in patients with advanced HCC.^[12]^ In addition, the combination treatment of TACE plus camrelizumab and apatinib showed significantly better overall survival, progression-free survival, and ORR compared to TACE monotherapy for predominantly advanced HCC.^[13]^ In order to summarize the treatment effects of HCC by local therapy (TACE or HAIC) combined with an immune checkpoint inhibitor plus an anti-angiogenic agent, this meta-analysis provides a reference for clinicians to make the best choice in clinical practice.

## 2. Materials and methods

### 2.1. Publication search

This meta-analysis was performed according to the Preferred Reporting Items for Systematic Reviews and Meta-Analyses statement,^[14]^ which was also registered at http://www.crd.york.ac.uk/PROSPERO/ (Review registry number: CRD42024554854). Two authors searched databases independently, including PubMed, Web of Science, Embase, Cochrane Library, China National Knowledge Infrastructure, and Wan Fang databases. The time range was from inception to August 1st, 2025. Any disagreement regarding the study was resolved through discussion by all authors. The team applied the Medical Subject Headings (MeSH) terms and free-text keywords for database searching: “Hepatocellular Carcinomas” [Mesh terms]; “Chemoembolization Therapeutic” [MeSH terms]; hepatic arterial infusion chemotherapy; “Immunotherapy” [MeSH terms]; Molecular Targeted Therapy [MeSH terms], Below is a description of the PubMed search methodology:

#1 “Hepatocellular Carcinomas” [Mesh terms]

#2 Liver Neoplasm OR Hepatic Neoplasms OR Hepatic Neoplasm OR Cancer of the Liver

OR Hepatocellular Cancer OR Hepatic Cancer OR Liver Cancer OR Liver Cancers OR Cancer of the Liver OR Cancer, Hepatocellular

#3 #1 OR #2

#4 “Chemoembolization, Therapeutic”[MeSH terms]

#5 Embolotherapy OR Embolotherapies OR Therapeutic Embolization OR Embolizations, Therapeutic OR Therapeutic Embolizations OR Transcatheter Arterial Embolization Chemotherapy OR Transcatheter Hepatic Artery Chemoembolization OR Transcatheter Arterial Embolization Chemotherapy OR Embolization Therapy OR Transhepatic Arterial Chemoembolization OR TACE

#6 #4 OR #5

#7 HAIC OR (Hepatic Arterial Infusion Chemotherapy)

#8 “Immunotherapy”[MeSH terms]

#9 Pembrolizumab OR Toripalimab OR Sintilimab OR Tislelizumab OR Camrelizumab OR

Atezolizumab OR Sugemalimab OR Nivolumab OR Penpulimab OR Serplulimab OR Immune Checkpoint Inhibitor OR PD-1 OR PD-L1

#10 #8 OR #9

#11 Molecular Targeted Therapy [MeSH terms]

#12 Tyrosine Kinase Inhibitor OR TKI OR Lenvatinib OR Sorafenib OR Regorafenib OR Cabozantinib OR Apatinib OR Donafenib OR Targeted Agent OR Anti-Angiogenic Therapy

# 13 #11 OR #12

# 14 #3 AND (#6 OR #7) AND #10 AND #13

### 2.2. Inclusion and exclusion criteria

#### 2.2.1. Inclusion criteria

The following were inclusion criteria: patients suffering from HCC confirmed via either pathological or clinical diagnosis regardless of gender, race, or age; the Local-TKIs-ICIs group received TACE or HAIC plus TKIs and ICIs treatment, while the TKIs-ICIs group only received TKIs and ICIs therapy; research design: the research was either retrospective or prospective; the language was limited to Chinese and English; clinical outcomes: ORR, disease control rate (DCR), median progression-free survival (mPFS), median overall survival (mOS), and treatment-related adverse events (TRAEs).

### 2.3. Exclusion criteria

Reviews, case reports, conference abstracts, and repeated studies; single-arm studies; studies with incomplete data and unable to obtain original data; and overlapping publications or those involving animal or cell experiments.

### 2.4. Data extraction and quality assessment

Two authors (Tao Sheng and Ying Tang) independently screened, extracted, and cross-checked the data. Disagreements were resolved through discussion or consultation with a third author (Shaohua Zhang). Extracted information: author, year, location, design, baseline characteristics, sample size, interventions, and outcomes. The included studies were retrospective; therefore, we chose the Newcastle-Ottawa Scale to evaluate the methodological quality of included studies.^[15]^ The total maximum score is 9 points. Studies achieving a score of 7 to 9 points were considered to be of high quality, whereas those with a score of 6 points or less were defined as moderate or low quality. See Table 1.

**Table 1:** Quality assessment of the enrolled retrospective cohort studies according to the Newcastle-Ottawa Scale.

Assessment indicators		①	②	③	④	⑤	⑥	⑦	⑧	⑨	⑩	⑪	⑫	⑬	⑭	⑮
Selection	Adequacy of exposed cohort selection	1	1	1	1	1	1	1	1	1	1	1	1	1	1	1
	Feasibility of nonexposed cohort selection	1	1	1	1	1	1	1	1	1	1	1	1	1	1	1
	Reliability of exposure confirmation	1	1	0	1	1	1	1	1	1	1	1	1	1	1	1
Comparability	No target outcome at study initiation	1	1	1	1	1	1	1	1	1	1	1	1	1	1	1
	Control for confounding factors	2	2	2	1	2	2	1	2	1	2	2	2	2	1	2
Outcome	Valid evaluation of research outcomes	1	1	1	1	1	1	1	1	1	1	1	1	1	1	1
	Sufficient follow-up period	1	1	1	1	1	1	1	1	1	1	1	1	1	1	1
	Low loss rate during follow-up	1	1	0	1	1	1	0	1	0	1	1	1	1	1	0
Total score		9	9	8	9	8	9	7	8	7	9	9	8	9	8	8

① Diao LF 2024, ② Mei J 2021, ③ Chen S 2023, ④ Jin ZC 2024, ⑤ Fu YZ 2023, ⑥ Guan RG 2024, ⑦ Chen S 2021, ⑧ Zhang JX 2023, ⑨ Huang JT 2022, ⑩ Wang JF 2023, ⑪ Zhang JX 2024, ⑫ Xin YJ 2023, ⑬ Chang X 2024, ⑭Wu CX 2025, ⑮ Yao Y 2025.

### 2.5. Statistical analysis

All statistical analyses in this meta-analysis were performed by Stata 17 (stataCorp LLC). We utilized odds ratios (OR) to evaluate dichotomous variables (ORR, DCR) and hazard ratios (HR) to assess continuous survival data (mOS, mPFS), both reported with 95% confidence interval (CI). Heterogeneity assessment included chi-square test and I^2^ test. When *I*^2^ ≤ 50% or *P* ≥ .05, indicating low heterogeneity, the combined proportion and 95% CI are calculated by a fixed-effect model. Conversely, the random effects model was considered (*I*^2^ > 50% or *P* < .05) for subgroup analyses according to different local treatment methods of TACE or HAIC. For all results, the difference reached statistical significance (*P* < .05).

## 3. Results

### 3.1. Search results

**Figure 1.**
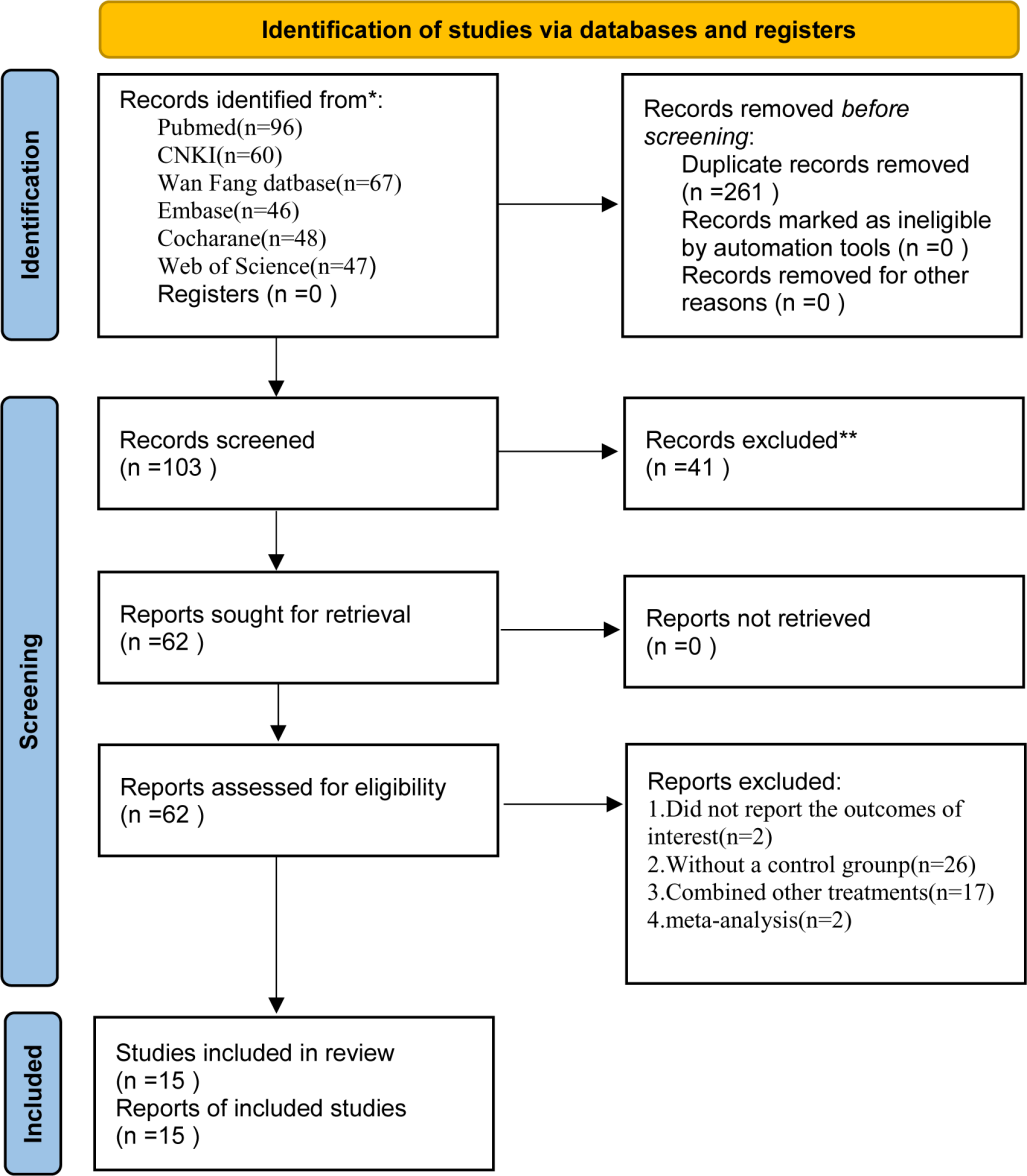
PRISMA flow diagram showing the screening and selection process. CNKI = China National Knowledge Infrastructure, n = number of studies, PRISMA = Preferred Reporting Items for Systematic Reviews and Meta-Analyses.

Our initial search found a total of 364 studies using an electronic database. After reading the titles, we removed duplicates. Only 103 were left; after screening, we removed 47 irrelevant cases and the other articles, for example, case reports, reviews, and conference reports. We read the full-text articles, and 62 were included, excluding 28 articles without a control group, 16 articles that combined other treatments, 1 article that was a meta-analysis, and 2 articles that did not report the outcomes of interest. Ultimately, only 15 articles were included,^[16–30]^, which came from China, and all were retrospective studies, involving a total of 3208 patients. The retrieval process is shown in Figure 1. The characteristics of the patients in the studies are depicted in Table 2.

**Table 2:** The included studies of the trial.

Study	Study type	Intervention	Sample size	Gender (M/F)	Age (Yr)	BCLC stage (B/C)	Child-Pugh class (A/B)	Endpoint
Diao LF 2024^[16]^	RCS	HAIC-L-C, P, T	58	49/9	52.3 ± 17.3	24/34	49/9	①②③④
		vs L-C, P, T	63	50/13	53.2 ± 15.6	25/38	55/8	
Mei J 2021^[17]^	RCS	HAIC-L-N, K, T, S	45	38/7	49.1 ± 10.6	5/40	44/1	①②③④
		Vs L-K, T, S	25	18,/7	50.1 ± 12.3	3/22	22/3	
Chen S 2023^[18]^	RCS	TACE-L-T	50	42/8	56 (43–62)	NA	36/14	①②③④
		vs L-T	50	46/4	55 (36–71)	NA	35/15	
Jin ZC 2024^[19]^	RCS	TACE-S, L, D, Ap, B-A, C, S, P, N, T	802	683/119	54 (48–63)	NA	659/143	①③④
		vs S, L, D, Ap, B-A, C, S, P, N, T	442	384/58	56 (48–62)	NA	360/82	
Fu YZ 2023^[20]^	RCS	HAIC-L-P, S, To, C, Ti	53	35/18	57 (45–62)	40/13	37/16	①②③④
		vs L-P, S, To, C, Ti	89	62/27	54 (53–60)	45/65	62/93	
Guan RG 2024^[21]^	RCS	HAIC-Len-P	127	91/26	52 ± 11.23	NA	85/42	①②③④
		vs Len-P	103	87/39	54 ± 11.54	NA	84/19	
Chen S 2021^[22]^	RCS	HAIC-L-P	84	72/12	52 (42–67)	22/62	71/13	①②③④
		vs P-L	86	71/15	53 (46–69)	21/65	75/11	
Zhang JX 2023^[23]^	RCS	TACE-L, So-Si, C	54	46/8	60 ± 17.46	23/31	NA	①②④
		vs L, So-Si, C	54	47/7	63 ± 14.32	21/33	NA	
Huang JT 2022^[24]^	RCS	TACE-So, L-Si, C	24	20/4	58.0 ± 10.7	NA	18/6	①②④
		vs So, L-Si, C	40	35/5	57.8 ± 13.1	NA	28/12	
Wang JF 2023^[25]^	RCS	TACE-L-Si, P	46	41/5	55 ± 11.92	8/38	38/8	①②③④
		vs L-Si, P	59	51/8	58 ± 9.82	9/50	45/14	
Zhang JX 2024^[26]^	RCS	TACE-L, So-C, Si, A	106	14/92	62 ± 10.31	NA	69/37	①②③④
		vs L, So-C, Si, A	109	8/70	65 ± 9.24	NA	52/22	
Xin YJ 2023^[27]^	RCS	TACE-L-T, C, Si	60	54/6	57 (26–76)	21/39	NA	①②③④
		vs L-T, C, Si	58	51/7	54 (28–78)	23/35	NA	
Chang X 2024^[28]^	RCS	HAIC-L-Si, C, T	103	91/12	52 ± 8.82	4/99	84/19	①②③④
		vs L-Si, C, T	61	57/4	56 ± 7.88	2/59	50/11	
Wu CX 2025^[29]^	RCS	HAIC-L-C, T, Si, T, P	158	142/16	62 ± 15.21	NA	134/24	①②③④
		vs L-C, T, Si, T, P	78	73/5	NA	NA	63/15	
Yao Y 2025^[30]^	RCS	TACE-L-T, C, Si	52	47/5	52.±10.21	NA	47/5	①②③④
		vs L-T, C	69	60/9	57.±13.45	NA	54/15	

① = ORR, ② = DCR, ③ = mPFS, ④ = mOS.

Ap = apatinib, B = bevacizumab, C = camrelizumab, D = donafenib, DCR = disease control rate, F = female, HAIC = hepaticarterial infusion chemotherapy, L = lenvatinib, M = male, mOS = median overall survival, mPFS = median progression-free survival, N = nivolumab, NA = not available, ORR = objective response rate, RCS = retrospective cohort study, Si = Sintilimab, S_O_ = sorafenib, T = tislelizumab, TACE = transarterial chemotherapy, To = toripalimab.

### 3.2. ORR

**Figure 2.**
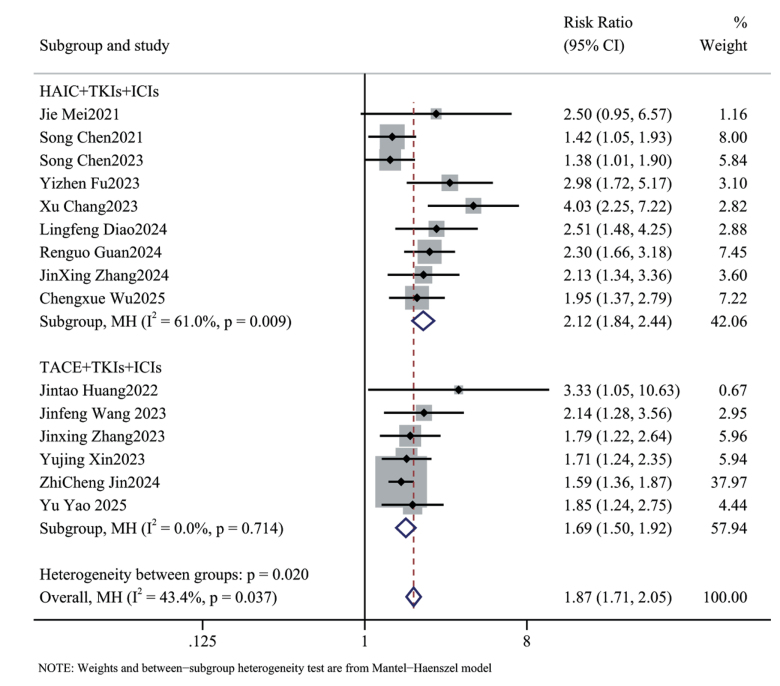
Meta-analysis results of the ORR in the TACE/ HAIC + TKIs + ICIs group vs TKIs + ICIs group for HCC. CI = confidence interval, HAIC = hepatic arterial infusion chemotherapy, HCC = hepatocellular carcinoma, ICI = immune checkpoint inhibitors, MH = Mantel Haenszel, ORR = objective response rate, TACE = transarterial chemoembolization, TKIs = tyrosine kinase inhibitors.

The overall effect showed a significant clinical advantage for the triple therapy group (relative risk [RR] = 1.87, 95% CI: 1.71–2.05, *P* < .05), with mild overall heterogeneity (*I*^2^ = 43.4%, *P* = .037). Nine studies were included in the HAIC subgroup; the pooled RR was 2.12 (95% CI: 1.84–2.44, *P* < .05), indicating a significant treatment benefit. Moderate to high heterogeneity (*I*^2^ = 61.0%, *P* = .009). In the TACE group (6 studies), the pooled RR was 1.69 (95% CI: 1.50–1.92, *P* < .05). This subgroup showed no detectable heterogeneity (*I*^2^ = 0.0%, *P* = .714), with highly stable results. The subgroup difference testing confirmed a statistically significant efficacy (*P* = .020). See Figure 2.

### 3.3. DCR

**Figure 3.**
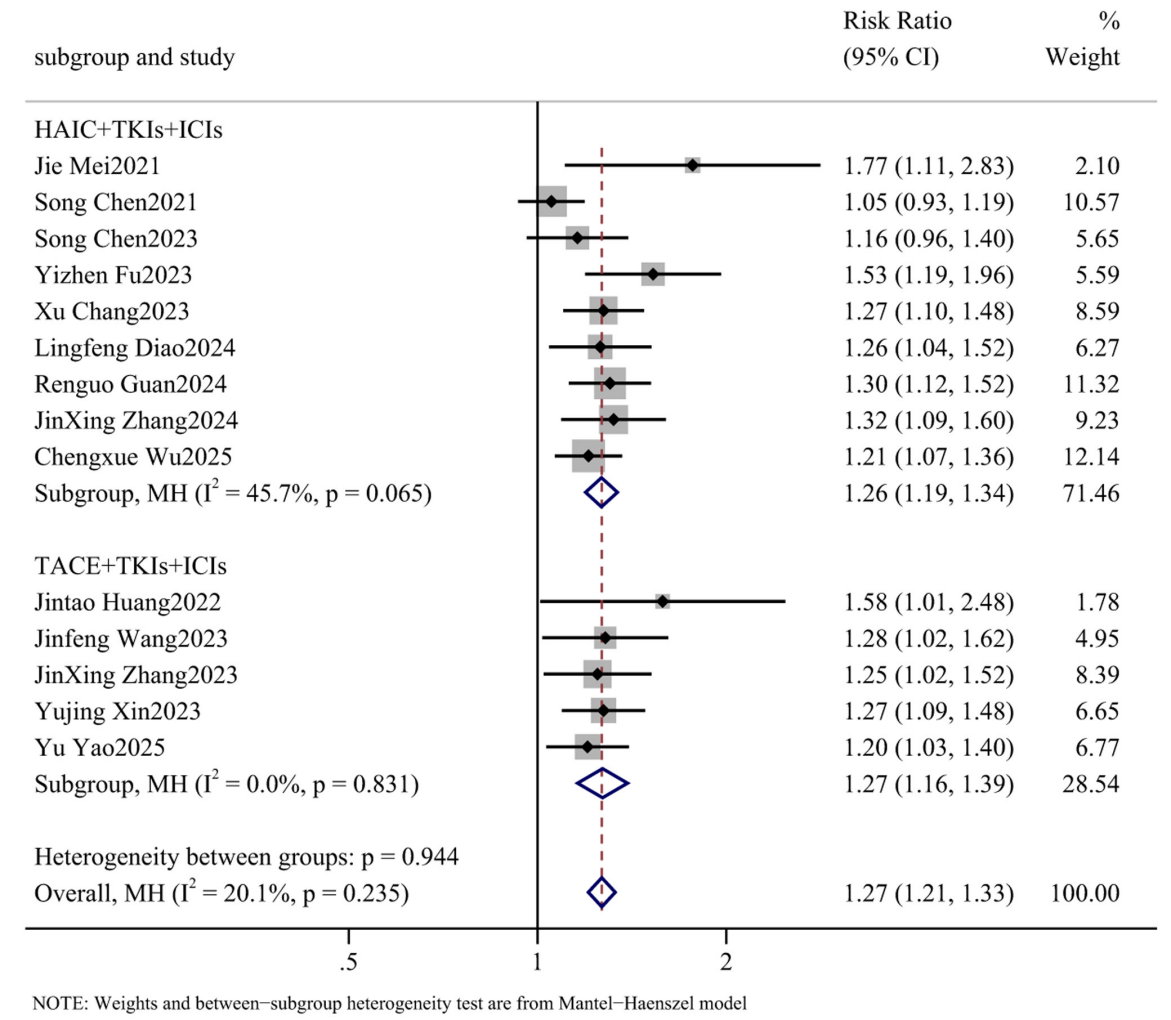
Meta-analysis results of the DCR in the TACE/ HAIC + TKIs + ICIs group vs TKIs + ICIs group for HCC. CI = confidence interval, DCR = disease control rate, HAIC = hepatic arterial infusion chemotherapy, HCC = hepatocellular carcinoma, ICI = immune checkpoint inhibitors, MH = Mantel Haenszel, TACE = transarterial chemoembolization, TKIs = tyrosine kinase inhibitors.

Including 14 research provided the result of DCR; the heterogeneity was low (*P* = .235, *I*^2^ = 20.1%). Fixed-effects model analysis indicated that the triple therapy group exhibited higher DCR compared to the dual regimen (RR = 1.27; 95% CI: 1.21–1.33; *P* = .000). The differences in DCR between the HAIC-TKIs-ICIs and TACE-TKIs-ICIs groups did not achieve statistically significant efficacy (RR = 1.26; 95% CI: 1.19–1.34; RR = 1.27; 95% CI: 1.16–1.39). See Figure 3.

### 3.4. mOS

**Figure 4.**
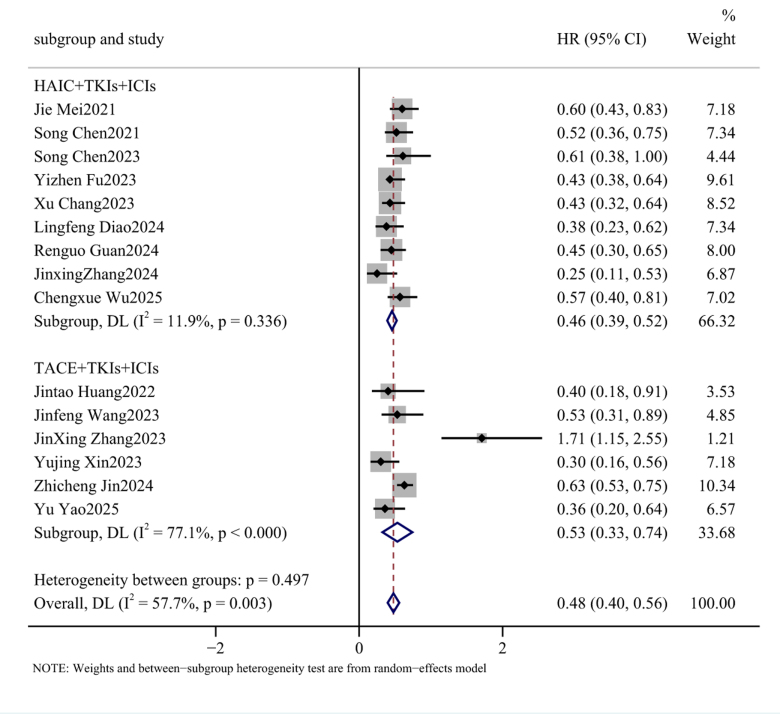
Meta-analysis results of the mOS in the TACE/ HAIC + TKIs + ICIs group vs TKIs + ICIs group for HCC. CI = confidence interval, DL = Dersimonian-Laird, HAIC = hepatic arterial infusion chemotherapy, HCC = hepatocellular carcinoma, HR = hazard ratio, ICI = immune checkpoint inhibitors, mOS = median overall survival, TACE = transarterial chemoembolization, TKIs = tyrosine kinase inhibitors.

Fifteen articles recorded the results of mOS. Overall heterogeneity indicated moderate heterogeneity (*I*^2^ = 57.7%, *P* = .003). Therefore, a random effects model was used for analysis. Both combination strategies showed prominent survival benefits for prolonging mOS (HR = 0.48; 95% CI = 0.40–0.56; *P* < .05). Subgroup analysis revealed that the HAIC-TKIs-ICIs group had a pooled HR = 0.46 (95% CI, 0.39–0.52) with heterogeneity (*I*^2^ = 11.9%, *P* = .336). The TACE-TKIs-ICIs subgroup achieved a pooled HR of 0.53 (95% CI, 0.33–0.74), with marked high heterogeneity (*I*^2^ = 77.1%, *P* < .001). Between subgroup comparison revealed no significant difference in therapeutic effect (*P* = .497). See Figure 4.

### 3.5. mPFS

**Figure 5.**
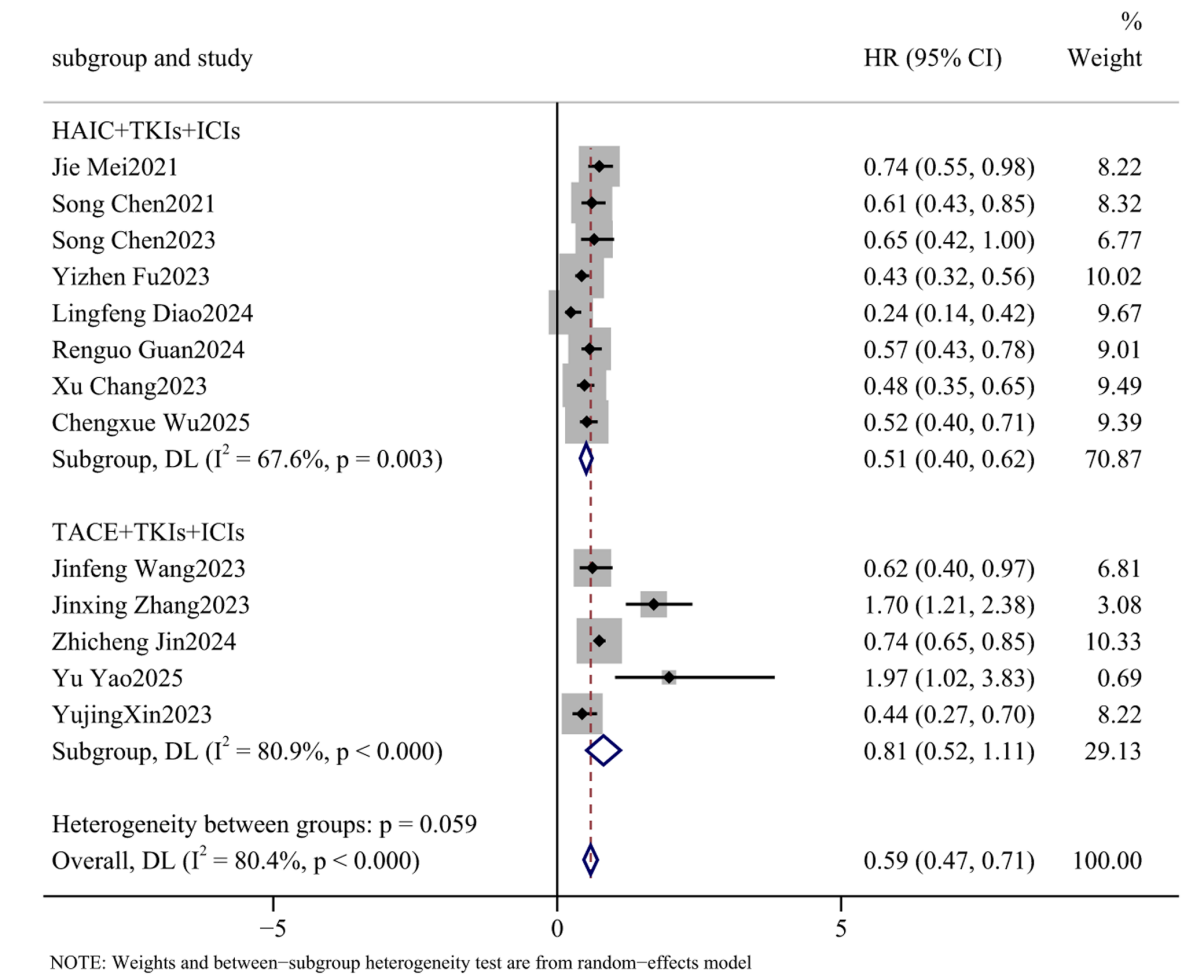
Meta-analysis results of the mPFS in the TACE/ HAIC + TKIs + ICIs group vs TKIs + ICIs group for HCC. CI = confidence interval, DL = Dersimonian-Laird, HAIC = hepatic arterial infusion chemotherapy, HCC = hepatocellular carcinoma, HR = hazard ratio, ICI = immune checkpoint inhibitors, mPFS = median progression-free survival, TACE = transarterial chemoembolization, TKIs = tyrosine kinase inhibitors.

The heterogeneity test results showed significant large heterogeneity among studies (*I*^2^ = 80.4%, *P* < .000). Random effects model analysis showed that the mPFS for patients treated with Local-TKIs-ICIs was longer compared to those treated with TKIs-ICIs (HR = 0.59; 95% CI: 0.47–0.71; *P* < .001). The differences comparing TACE-TKIs-ICIs with HAIC-TKIs-ICIs were not statistically significant (HR = 0.81; 95% CI: 0.52–1.11; HR = 0.51; 95% CI: 0.4–0.62). See Figure 5.

### 3.6. Assessment of publication bias

**Figure 6.**
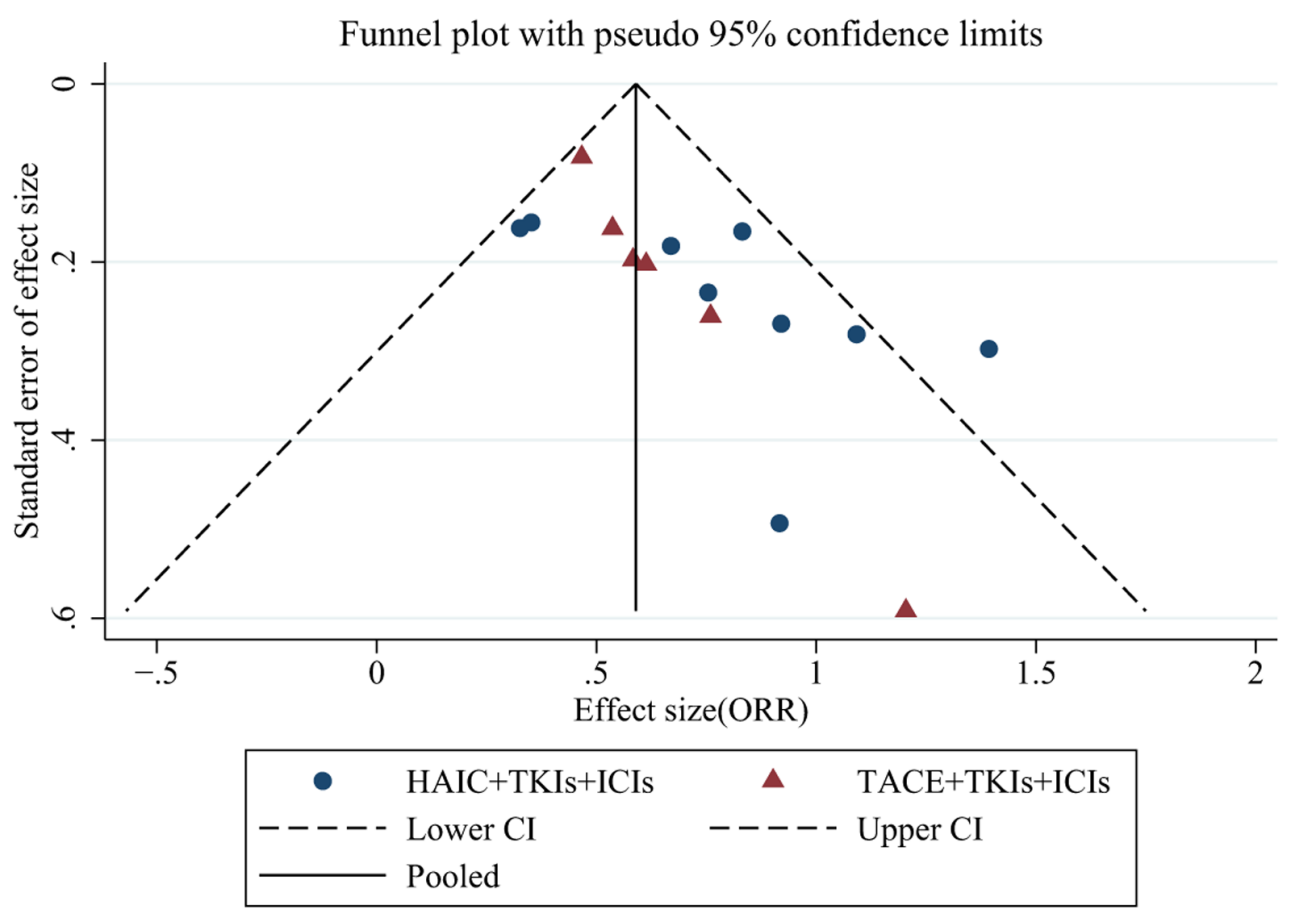
The funnel plot of the median ORR. CI = confidence interval, HAIC = hepatic arterial infusion chemotherapy, HCC = hepatocellular carcinoma, ICI = immune checkpoint inhibitors, ORR = objective response rate, TACE = transarterial chemoembolization, TKIs = tyrosine kinase inhibitors.

**Figure 7.**
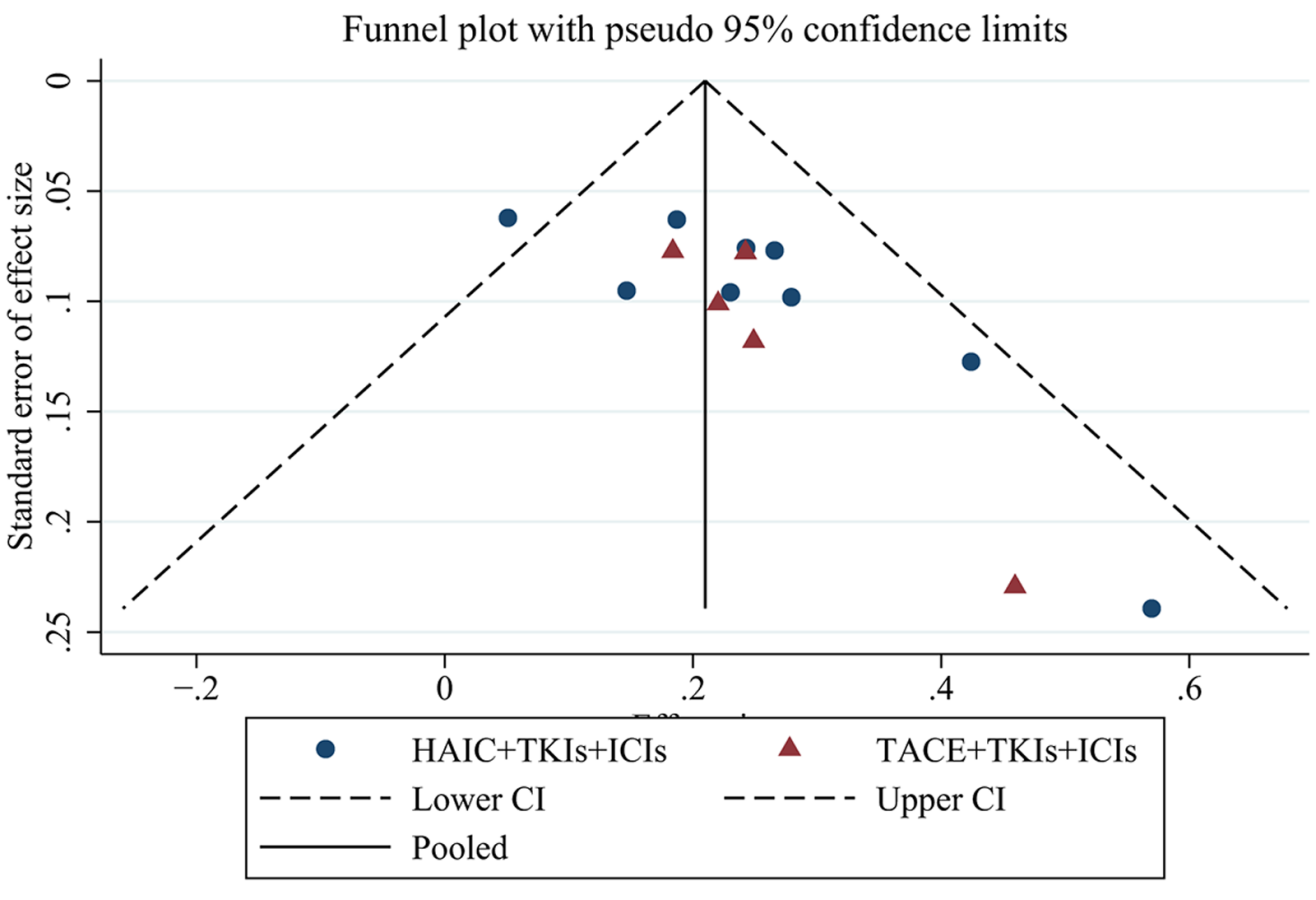
The funnel plot of the DCR. CI = confidence interval, DCR = disease control rate, HAIC = hepatic arterial infusion chemotherapy, HCC = hepatocellular carcinoma, ICI = immune checkpoint inhibitors, TACE = transarterial chemoembolization, TKIs = tyrosine kinase inhibitors.

**Figure 8.**
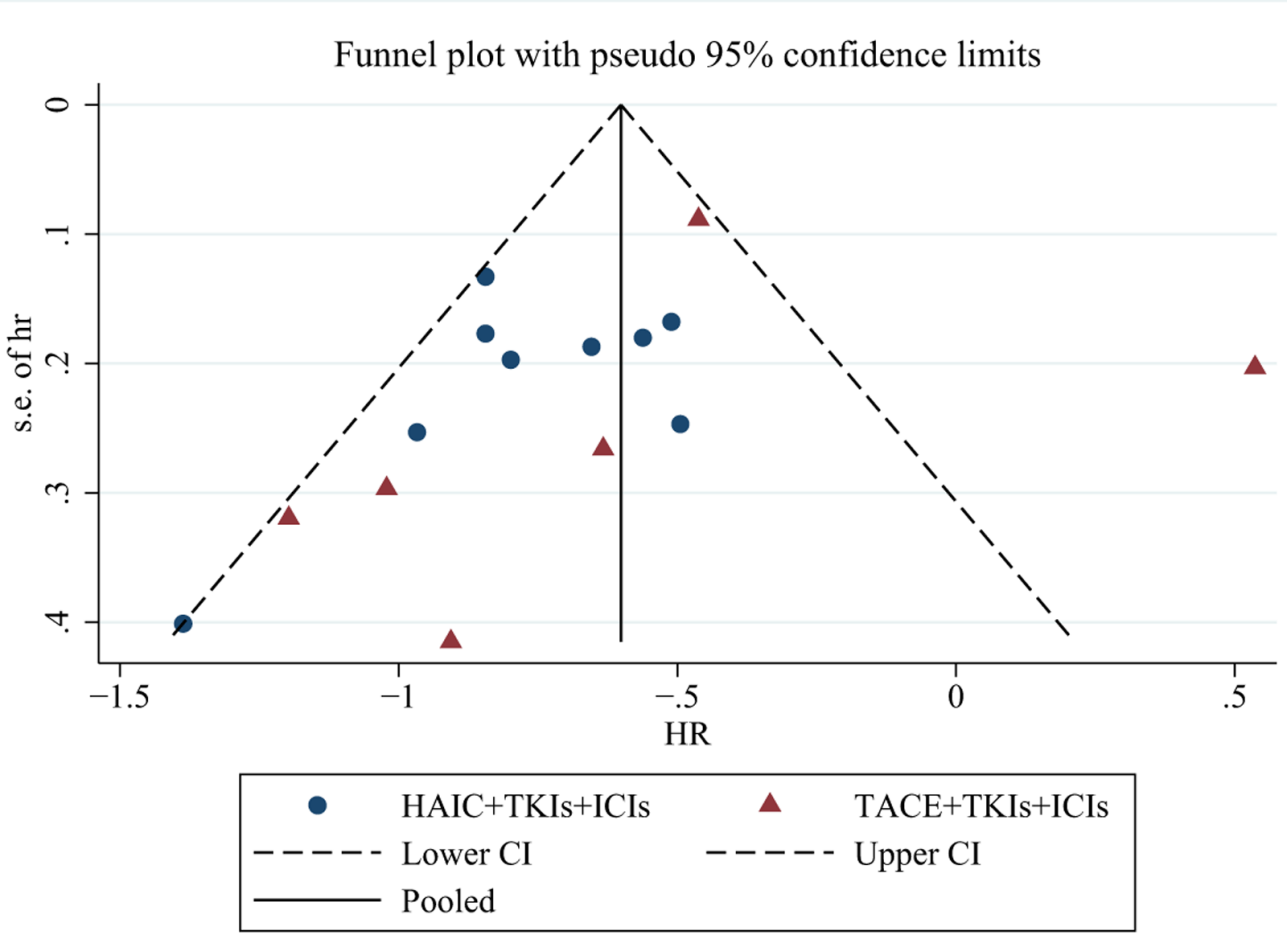
The funnel plot of the mOS. CI = confidence interval, HAIC = hepatic arterial infusion chemotherapy, HCC = hepatocellular carcinoma, HR = hazard ratio, ICI = immune checkpoint inhibitors, mOS = median overall survival, SE = standard error, TACE = transarterial chemoembolization, TKIs = tyrosine kinase inhibitors.

**Figure 9.**
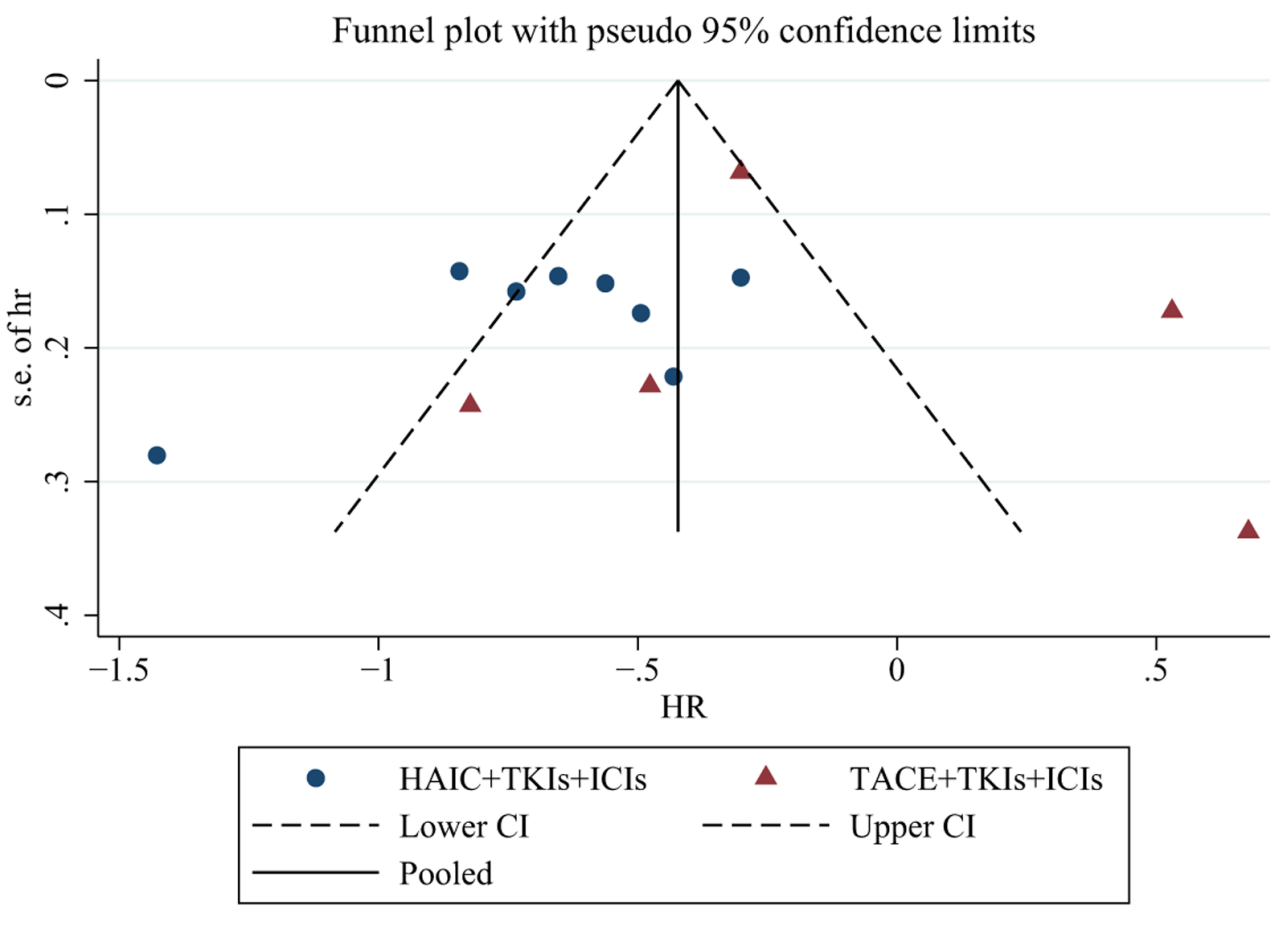
The funnel plot of the mPFS. CI = confidence interval, HAIC = hepatic arterial infusion chemotherapy, HCC = hepatocellular carcinoma, HR = hazard ratio, ICI = immune checkpoint inhibitors, mPFS = median progression-free survival, SE = standard error, TACE = transarterial chemoembolization, TKIs = tyrosine kinase inhibitors.

Funnel plots of ORR and DCR showed well symmetry, indicating no significant publication bias for short-term response endpoints. In comparison, mOS and mPFS funnel plots presented mild asymmetry, suggesting slight potential publication bias for survival outcomes. Subgroup distribution was more balanced in the HAIC-TKIs-ICIs group, while greater dispersion was seen in the TACE-TKIs-ICIs cohort, consistent with its high statistical heterogeneity. In general, the overall results of this meta-analysis are reliable, and the minor survival-related bias does not overturn the main therapeutic conclusions. See Figure 6. Figure 7, Figure 8, Figure 9.

### 3.7. TRAEs

The incidence rates of TRAEs were presented in Tables 3 and 4. In comparison with TKIs-ICIs alone, Local-TKIs-ICIs significantly elevated the incidence of all-grade abdominal pain, appetite loss, nausea, leukopenia, and aspartate aminotransferase (AST) elevation while decreasing the risk of hypothyroidism (*P* < .05). Meanwhile, the triple therapy increased the incidence of grade 3 to 4 adverse events of abdominal pain and liver injury. Conversely, there was no significant difference between the 2 treatment groups in the incidence of diarrhea, hand-foot skin reaction, fatigue, and hypertension (*P* > .05).

**Table 3:** The TRAEs of meta-analysis (any grade).

TRAEs	Trials	Heterogeneiy *P*, *I*^2^ (%)	Meta-analysis RR, 95% CI, *P*	Subgroup (TACE) RR, 95% CI, *P*	Subgroup (HAIC) RR, 95% CI, *P*
Diarrhea	13	.788, 8.8	0.93 (0.76, 1.12), .357	0.94 (0.74, 1.21), .445	0.89 (0.64, 1.24), .231
Hand-foot skin reaction	11	.432, 70	1.34 (1.03, 1.40), .233	1.36 (0.95, 1.92), .071	1.30 (0.96, 1.70), .054
Abdominal pain	11	.001, 86.6	3.53 (2.86, 4.37), .000	2.20 (1.6, 3.04), .007	4.5 (3.4, 5.97), .000
Fatigue	13	.733, 73	1.12 (0.95, 1.32), .373	1.14 (0.93, 1.14), .083	1.08 (0.83, 1.4), .796
Nausea	8	.003, 6.8	1.45 (1.14, 1.83), .002	2.3 (1.56, 3.62), .721	1.16 (0.45, 3), .000
Decreased Appetite	10	.00, 74.8	3.43 (2.56, 4.37), .001	2.57 (1.7, 3.9), .000	1..08 (0.85, 1.37), .535
Hypothyroidism	13	.047, 66	0.78 (0.64, 0.96), < .000	0.99 (0.71, 1.36), .354	0.64 (0.49, 0.84), .428
Leukopenia	5	.32, 23	2.03 (1.52, 2.71), .000	2.07 (1.54, 2.78), .000	1.33 (0.31, 5.65), .6964
Aspartate aminotransferase	11	.21, 73.3	1.35 (1.19, 1.52), .000	1.72 (1.35, 2.19), .005	1.19 (1.04, 1.37), .008
Hypertension	13	.345, 61.8	0.94 (0.83, 1.07), .02	1.02 (0.86, 1.21), .378	0.85 (0.70, 1.02), 0.001

CI = confidence interval, HAIC = hepatic arterial infusion chemotherapy, RR = relative risk, TACE = transarterial chemoembolization, TRAE = treatment-related adverse event.

**Table 4:** The TRAEs of meta-analysis (3–4 grade).

TRAEs	Trails	Heterogeneity *P*, *I*^2^ (%)	Meta-analysis RR, 95% CI, *P*	Subgroup (TACE) RR, 95% CI, *P*	Subgroup (HAIC) RR, 95% CI, *P*
Diarrhea	11	.77, 0.00	1.39 (0.78, 2.51), .267	1.97 (0.84, 4.44), .102	0.9 (0.37, .2.18), .813
Hand-foot skin reaction	7	.32, 70	1.04 (0.98, 1.40), .233	1.25 (0.93, 1.92), .075	1.10 (0.87, 1.50), .54
Abdominal pain	6	.08, 49.5	3.12 (1.90, 5.13), .000	1.48 (0.57, 3.83), .424	3.95 (2.18, 7.18), .000
Fatigue	5	.21, 0.0	1.12 (0.58, 2.15), .745	1.57 (0.66, 3.76), .309	0.65 (0.22, 1.87), .422
Nausea	3	.02, 0.00	1.75 (0.95, 3.19), .071	-	1.64 (0.89, 2.99), .11
Decreased appetite	5	.76, 0.00	1.58 (0.74, 3.38), .24	2.0 (0.37, 10.75), .419	1..48 (0.63, 3.49), .367
Hypothyroidism	7	.85, 0.00	0.86 (0.44, 1.68), .658	0.75 (0.14, 3.93), .731	0.89 (0.43, 1.89), .747
Leukopenia	4	.51, 23	1.03 (0.52, 2.71), .121	2.07 (0.98, 2.78), .215	1.35 (0.51, 5.13), .6964
Aspartate aminotransferase	10	.07, 49.5	2.22 (1.57, 3.14), .000	3.05 (1.8, 5.18), .043	1.61 (1.02, 2.54), .000
Hypertension	9	.98, 0.00	0.97 (0.7, 1.33), .834	0.97 (0.64, 1.46), .882	0.96 (0.58, 1.59), .881

CI = confidence interval, HAIC = hepatic arterial infusion chemotherapy, RR = relative risk, TACE = transarterial chemoembolization, TRAE = treatment-related adverse events.

## 4. Discussion

In recent years, systematic treatment strategies for HCC have garnered extensive attention. Local interventional therapies, including TACE and HAIC, alongside targeted therapy and immunotherapy, combining 2 or more approaches, have shown promising results. TACE is expected to effectively kill HCC lesions. Under the premise of controlling side effects, the quality of life of patients is greatly improved, and the life of patients is prolonged.^[31]^ Some studies have explored the disparities in long-term clinical efficacy and tolerability between the combination of TACE with TKIs and ICIs (TACE-TKIs-ICIs) and TKIs-ICIs alone.^[32]^ Up to the present, no consistent clinical guidelines have been reached for HCC management, which stems primarily from the significant heterogeneity of these patients. Our study evaluated the differences between these 2 therapy modalities, with the aim of providing a more reliable basis for the selection of HCC treatments.

In this research, we conducted a comprehensive evaluation of 15 retrospective studies, encompassing a total of 3208 individuals. Pooled effect estimates consistently indicated that regimens combining TACE or HAIC with TKIs and ICIs exhibited remarkable efficacy endpoints, including ORR, DCR, mPFS, and mOS. In the present meta-analysis, overall survival was prospectively defined as the critical endpoint. The results confirm that triple combination therapy confers a substantial and statistically significant survival benefit, prolonging the mOS in the target patient population. Additionally, comparative analysis further revealed that triple treatment experienced a significantly extended mPFS compared to those receiving dual-agent therapy (RR = 0.59; 95% CI: 0.47–0.71).

The triple-combined strategy demonstrates a significant synergistic effect. Interventional therapies of TACE and HAIC induced primary tumor necrosis, triggering Immunogenic Cell Death. This massive release of tumor-associated antigens effectively “hot-wires” the immune system, converting “cold” tumors into “hot” ones by recruiting tumor-infiltrating lymphocytes to the site.^[33]^ Further enhancing the antitumor immune response elicited by PD-1 blockade. TKIs exert potent inhibitory effects on tumor proliferation and pathological angiogenesis, alleviating the hypoxia-dependent vascular proliferation induced by interventional therapies. Targeted drugs can reshape the immunosuppressive state of the tumor microenvironment and substantially enhance the immunotherapeutic efficacy of PD-1 inhibitors in HCC. The complementary effects among different treatment modalities collectively exert potent antitumor activity, which further improves the long-term clinical prognosis of HCC patients.^[34,35]^ PD-1 inhibitors exert notable immune-regulatory effects. Interventional therapies can effectively reduce the overall tumor burden, increase the infiltration level of intratumoral cluster of differentiation 8 positive T lymphocyte (CD8^+^T cells), and reverse the immune dysfunction of cytotoxic T cells. Triple therapy triggers robust local and systemic immune responses, achieving sustained survival benefits in the HCC population.^[36]^

Common TRAEs may be correlated with TKIs and ICIs and were detected, including hypertension, hand-foot skin reactions, and fatigue. Compared with the group receiving TKIs-ICIs alone, the Local-TKIs-ICIs group significantly increased the incidence of all-grade abdominal pain, appetite loss, nausea, leukopenia, and AST elevation, while reducing the risk of hypothyroidism (*P* < .05). Subgroup analysis reveals that HAIC significantly increases abdominal pain, but TACE tends to cause nausea, decreased appetite, and leukopenia. The triple therapy raises grade 3 to 4 abdominal pain and AST elevation. Both treatment regimens predominantly presented mild-to-moderate adverse events, demonstrating favorable safety and tolerability. In the selection of treatment methods for patients, clinicians need to conduct a preoperative comprehensive assessment of organ function and underlying diseases, and preventive interventions should be administered to high-risk patients. Medication methods are individually adjusted through dynamic monitoring to promptly optimize strategies, bringing well therapeutic effects and minimal side effects to patients.

This research encompasses several restrictive factors. Firstly, certain outcome measures exhibited evident heterogeneity, and more influencing factors need to be identified. Secondly, the research individuals belong to the Chinese population, and the findings may not be generalizable to other countries. Thirdly, the average number of subjects recruited in each study was relatively small, and no multicenter randomized controlled trial was incorporated into this meta-analysis. In future research, we need to carry out much larger-scale, multicenter randomized controlled trial studies.

In summary, TACE or HAIC combined with ICIs and TKIs significantly improved the ORR and DCR, prolonged the survival time of mOS and mPFS compared with the ICIs and TKIs therapy. Although combination therapy increases adverse reactions, most adverse reactions are controllable, and there are no serious adverse reactions leading to death. Clinically, further stratification is needed to determine which HCC patients should choose triple combination therapy.

## Author contributions

**Conceptualization:** Ying Tang.

**Data curation:** Ying Tang.

**Formal analysis:** Ying Tang.

**Funding acquisition:** Shaohua Zhang.

**Investigation:** Ying Tang, Xinbo Mao, Tao Sheng.

**Methodology:** Xinbo Mao.

**Supervision:** Shaohua Zhang.

**Validation:** Shaohua Zhang.

**Visualization:** Shaohua Zhang.

**Writing – original draft:** Shaohua Zhang.

**Writing – review & editing:** Shaohua Zhang, Tao Sheng.
